# A re-assessment of the safety of silver in household water treatment: rapid systematic review of mammalian in vivo genotoxicity studies

**DOI:** 10.1186/s12940-017-0279-4

**Published:** 2017-06-20

**Authors:** Lorna Fewtrell, Batsirai Majuru, Paul R. Hunter

**Affiliations:** 1 Centre for Research into Environment and Health, University of Aberystwyth, Aberystwyth, UK; 2 Norwich Medical School, University of East Anglia, Norwich, NR4 7TJ UK

**Keywords:** Silver, Nanoparticles, Genotoxicity, DNA damage

## Abstract

**Background:**

Despite poor evidence of their effectiveness, colloidal silver and silver nanoparticles are increasingly being promoted for treating potentially contaminated drinking water in low income countries. Recently, however, concerns have been raised about the possible genotoxicity of particulate silver.

**Objectives:**

The goal of this paper was to review the published mammalian in vivo genotoxicity studies using silver micro and nanoparticles.

**Methods:**

SCOPUS and Medline were searched using the following search string: (“DNA damage” OR genotox* OR Cytotox* OR Embryotox*) AND (silver OR AgNP). Included papers were any mammalian in vivo experimental studies investigating genotoxicity of silver particles. Studies were quality assessed using the ToxRTool.

**Results:**

16 relevant papers were identified. There were substantial variations in study design including the size of silver particles, animal species, target organs, silver dose, route of administration and the method used to detect genotoxicity. Thus, it was not possible to produce a definitive pooled result. Nevertheless, most studies showed evidence of genotoxicity unless using very low doses. We also identified one human study reporting evidence of “severe DNA damage” in silver jewellery workers occupationally exposed to silver particles.

**Conclusions:**

With the available evidence it is not possible to be definitive about risks to human health from oral exposure to silver particulates. However, the balance of evidence suggests that there should be concerns especially when considering the evidence from jewellery workers. There is an urgent need to determine whether people exposed to particulate silver as part of drinking water treatment have evidence of DNA damage.

## Background

Household point-of-use (POU) treatment is being widely promoted as an interim measure to improve the quality of drinking water in low income settings where consumers do not have access to improved drinking water supplies. There is a range of different technologies that are being promoted by different agencies and non-governmental organizations that can be broadly categorised as disinfection or filtration technologies [1, 2]. Within this diverse range of technologies colloidal silver (Ag) is being promoted as a primary water disinfectant, both silver nitrate (AgNO_3_) and silver nanoparticles (AgNP) are being added to the surface of ceramics and AgNP is being investigated as an adjunct to filtration technologies in a wide number of experimental systems [3–5]. Typically, nanoparticles are small particles with one dimension being 100 nm or less. Colloidal Ag consists of micro and nanoparticles of silver, typically in the range of 2–500 nm, dispersed throughout another substance [6].

Among those promoting the use of colloidal Ag or AgNP there is the general assumption that this product is safe in humans. The World Health Organization (WHO) Guidelines for Drinking-water Quality currently do not have a guideline value for Ag in drinking water, but indicate a concentration of 0.1 mg/L which could be tolerated without risk to health [7]. However, this value was determined as being unlikely to cause argyria (a discolouring of the skin due to silver deposition) and took no consideration of possible adverse effects associated with AgNP, nor did it consider any potential genotoxicity.

Based on a study of a woman with argyria it was estimated that following consumption of silver acetate, up to 18% of the dose was absorbed [8]. From animal studies, AgNP adsorption is less than that for Ag salts, suggesting a lower bioavailability for AgNP [6].

After absorption Ag and AgNP are widely distributed through the body. Retention of Ag and AgNP varies between different organs, although retention in the brain and testes seems to be particularly strong [9].

A review of the safety of Ag, colloidal Ag and AgNP was published by Hadrup and Lam [6]. They reported that a range of dose-dependent toxic effects have been described in experimental animals including: “weight loss, hypoactivity, altered neurotransmitter levels, altered liver enzymes, altered blood values, enlarged hearts and immunological effects”. However, most of these effects were seen at doses well above levels likely to be consumed in water, even with the use of Ag as a primary water disinfectant. Nevertheless, they still recommended a Tolerable Daily Intake (TDI) value of 2.5 μg/kg of body weight/day.

An area of further interest is whether exposure to Ag and AgNP is associated with genotoxic effects. Hadrup and Lam concluded that Ag only has limited genotoxic effects [6]. Their conclusions, however, were based on a relatively small number of studies, two of which were concerned with Ag halide salts and two with AgNP. Neither of the two studies of Ag halides found evidence of genotoxicity. Of the two studies with AgNPs one found no micronucleus induction following 28 days of oral 60 nm silver nanoparticle administration [10]. The other study reported micronucleus induction in the TK6 lymphoblastoid cell line of rats following incubation with 5 nm silver nanoparticles at a concentration of 25 μg/mL [11].

In a substantially more detailed review of the genotoxicity of a wide range of nanoparticles, Magdolenova and colleagues included 13 different Ag microparticles and AgNP publications [12], of which one was an in vivo study [10]. Of the in vitro studies detailed, 9 used human cells or cell lines. A variety of Ag particle sizes (3 to >200 nm), coatings (including none, starch, polysaccharide and polyvinyl pyrrolidone), doses (0.01 μg/mL to 100 mg/mL), exposure periods and genotoxicity assay methods were used in the identified studies. Every in vitro mammalian cell-based study found AgNP to be genotoxic. Although many of the studies tested only relatively high concentrations of AgNP (up to 100 μg/mL), some studies used much lower concentrations and evidence of genotoxicity was found in human bronchial epithelial cells using both the micronucleus and comet assays at levels as low as 0.01 μg/mL [13].

Since the publication of these reviews, there has been a substantial number of papers published investigating the genotoxicity of silver AgNP in a wide variety of both in vivo and in vitro models. While the general consensus from the in vitro studies is that AgNP are genotoxic, the most important evidence would come from the in vivo studies. Thus, in order to investigate whether or not AgNP and, therefore, colloidal silver are likely to be genotoxic to humans and present a risk to health through its use in household drinking water treatment, we conducted a systematic review of in vivo mammalian studies administering AgNP and Ag microparticles.

## Methods

Searches were run in both SCOPUS and Medline using the following search string: (“DNA damage” OR genotox* OR Cytotox* OR Embryotox*) AND (silver OR AgNP). Initial searches were run on 15 November 2015 and then again on 29 February 2016. Searches were run without restrictions for date or language.

Inclusion criteria were any in vivo experimental study investigating genotoxicity of silver particles in mammalian models. In vitro studies in cell culture were excluded as were any in vivo studies in non-mammals such as fish, insects or helminths. In addition, we included any observational studies of genotoxicity in humans exposed to silver particles. Data were extracted from included papers into an excel spreadsheet by one author and checked by another. Data extracted included study identifier, year of publication, description of silver nanoparticles, the animal model used, the target organs, route of administration of nanoparticles, the study design, doses and frequency of administration, the time to a sacrifice after administration, method of detecting genotoxicity, whether genotoxic effects were identified and a short narrative summary of the study conclusions. The basic outcome measure was whether or not genotoxic effects had been identified.

The selected papers, which used either the comet assay or the micronucleus assay (both of which have Organisation for Economic Co-operation and Development [OECD] test guidelines) were subjected to a reliability assessment using ToxRTool [14], which results in each paper being scored out of 21 and the assignment of one of four categories (based on a previous paper by Klimisch and colleagues [15], namely:

1. Reliable without restrictions (score of 18 and above);
2. Reliable with restrictions (score of 13–17);
3. Not reliable (score of 12 and below);
4. Not assignable.

In order to be considered reliable (i.e. category 1 or 2) the minimum score must be achieved and the report must achieve all of the 8 ‘red’ (i.e. essential) criteria. The results are shown as the overall category followed by the score in brackets and, where applicable, the reason the study was downgraded to not reliable.

## Results

**Fig. 1:**
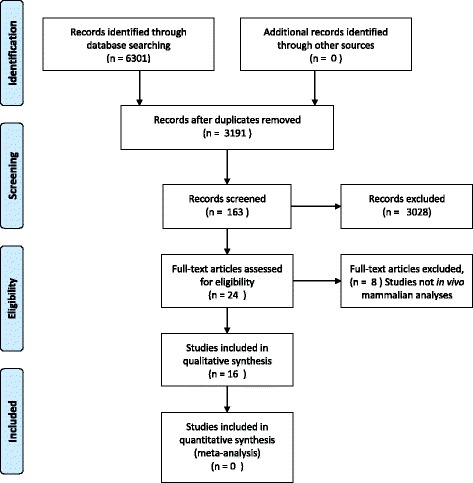
PRISMA Flow Diagram of in vivo mammalian genotoxicity studies of silver nanoparticles [34]

The initial search yielded 3191 references which were scanned by title and abstract. Of these references, 163 papers were identified for further study (Fig. 1). We finally identified 16 papers that investigated in vivo genotoxicity of silver nanoparticles, though one paper reported using both single and multiple dose study designs and we have considered these separately; making 17 studies in total. Those studies (15) reporting use of either the micronucleus assay or the comet assay are outlined in Table 1. An additional two studies are described in Table 2. The first paper identified was published in 2008 and the second in 2009 and then no more until 2011. There has been a substantial increased interest in this topic with half of the papers (8 of 16) being published from 2014 onwards.

**Table 1:** Characteristics of included studies, reporting use of micronucleus or comet assay, ordered by administration and study design

Identifier	Nanoparticles size & source/manufacture	Animal model	Target organ	Admin.	Study design	Dose administered	Time to sacrifice	Method	Genotoxic effect reported	Summary of results	Quality estimate
Al Gurabi 2015 [35]	mean 43.6 nm (reduction with aniline and CTAB)	Swiss albino mice	Peripheral blood	IP	Single dose	26, 52 & 78 mg/kg	24 and 72 h	Alkaline comet assay	y	Increased DNA strand breakages with all doses at 72 h post-exposure	3 (14) no +ve control; choice of frequency & duration of exposure not explained
Chen 2015 [18]	8 nm (poly styrene-co-maleic anhydride-coated)	Mice	Peripheral blood	IP	Single dose	0.25 & 1.0 mg/kg	48 & 72 h after injection	Micronucleus assay	n	No significant difference	3 (16) no –ve control, MN not scored in enough cells
Ghosh 2012 [36]	90–180 nm (Sigma-Aldrich)	Mice	Bone marrow	IP	Single dose	10, 20, 40 & 80 mg/kg body weight	18 h	Alkaline comet assay & microscopic examination of cells in metaphase	y	Significant increases seen in both the comet assay and chromosome aberration test at all doses. No clear dose–response relationship	2 (19) choice of frequency & duration of exposure not explained
Ordzhonikidze 2009 [16]	9 nm	Mice	Spleen	IP	Single dose	1.5 mg/kg	From 3 to 48 h	Neutral comet assay	n	A non-significant increase in DNA damage seen for both AgNP and the anionic surfactant used as a stabilizer	3 (13) no +ve control, use of neutral comet assay
Asare 2016 [37]	20 nm and 200 nm (PlasmaChem)	Mice	Lung, liver and testes	IV	Single dose	5 mg/kg	1 or 7 d post injection	Alkaline comet assay	n	No significant effect in any tissue (20 nm)	3 (18) no +ve control; time to sacrifice not ideal for comet assay
Dobrzyńska 2014 [38]	20 nm or 200 nm (PlasmaChem)	Rats	Bone marrow	IV	Single dose	5 & 10 mg/kg bw of 20 nm size AgNPs 5 mg/kg bw of 200 nm size AgNPs	24 h, 1 week and 4 weeks post-exposure	Alkaline comet and micronucleus assays	y	No effect seen in comet assay. Significantly increased frequency of erythrocyte micronuclei after 24 h of exposure for both doses. Enhanced frequency also seen at 1 and 4 weeks	3 (18) no +ve control
Gromadzka-Ostrowska 2012 [20]	20 & 200 nm (PlasmaChem)	male Wistar rats	Testes	IV	Single dose	5 & 10 mg/kg	24 h, 7 and 28 d post injection	Allkaline comet assay	y	Significant increased risk of DNA damage in spermatozoa seen 24 h post-exposure for 20 nm AgNP	3 (18) no +ve control
Tavares 2012 [17]	5–45 nm (citrate reduction)	Swiss mice	Peripheral blood	IV	Single dose	10, 25 & 50 μg/kg bw	1, 6, 12, and 24 h	Alkaline comet assay	n	Limited effects with only the lowest dose (after 24 h) producing a significant increase in DNA damage compared to the control	3 (17) no +ve control, no of animals/group not clearly stated
Kim 2011 [39]	18 nm ave. (naked)	Sprague-Dswley rats	Bone marrow	Inhalation	Repeated dose	30, 300 & 1000 mg/kg bw for 90 days	24 h	Micronucleus assay	n	No statistically significant differences were seen	3 (19) no +ve control
Awasthi 2015a [40]	5 nm	Swiss albino mice	Liver	Oral	Repeated dose	10 & 20 mg/kg bw once a week for 5 weeks	3 h after last dose	Alkaline comet assay	y	Significant DNA damage was seen at both doses	2 (17)
Awasthi 2015b [19]	10 nm	Swiss albino mice	Liver	Oral	Repeated dose	50 & 100 mg/kg bw on alternate days for 28 days	After exposure to the dosing regime	Alkaline comet assay	y	Dose-related increase in DNA damage seen, statistically significant at highest dose	3 (15) no +ve control
Kim 2008 [10]	60 nm (Nanatech Co. Ltd) CMC	Sprague–Dawley Rats	Bone marrow	Oral	Repeated dose	30, 300, and 1000 mg/kg/day for 28 days	24 h after last dose	Micronucleus assay	n	Non-significant increase in evidence of DNA damage with increasing dose	3 (19) no +ve control
Kovvuru 2015 [28]	5–150 nm (Sigma-Aldrich – PVP coated)	Mice	Offspring, peripheral blood, bone marrow	Oral	Repeated dose	500 mg/kg bw daily for 5 days	20 d for offspring, 24 h after first and last dose for blood and 24 h after last treatment for bone marrow	Micronucleus assay	y	Significant induction of micronuclei seen in blood (at 1 and 5 days) and bone marrow. Maternal ingestion of AgNP during gestation was found to induce large-scale genome rearrangements in developing embryos	3 (19) no +ve control
Patlolla 2015 [41]	10 nm (Ocean Nanotech)	Sprague–Dawley rats	Bone marrow	Oral	Repeated dose	5, 25, 50, 100, mg/kg bw, daily for five days	24 h after last dose	Comet and micronucleus assay	y	Significant effects seen in comet and micronucleus assays for doses of 50 mg/kg bw and above	3 (20) no +ve control
Awasthi 2015a [40]	5 nm	Swiss albino mice	Liver	Oral	Single dose	50 & 100 mg/kg bw	3 and 24 h after dose	Comet assay	y	Significant difference in all comet assay parameters (3 & 24 h) for highest dose	2 (17)

*CTAB* cetyltrimethylammonium bromide, *PVP* polyvinylpyrrolidone, *CMC* carboxymethylcellulose

**Table 2:** Characteristics of included studies using tests other than micronucleus and comet assay

Identifier	Nanoparticles	Animal model	Target organ	Admin.	Study design	Dose administered	Time to sacrifice	Method	Genotoxic effect reported	Summary of results
El Mahdy [42]	mean 8.7 nm (chemical reduction – PVP stablization)	Albino rats	Liver	IP	Repeated dose	1, 2 and 4 mg/kg bw daily for28 days	At end of study	Microscopic examination of cells in metaphase	y	Increased chromosomal aberrations, significant at 2 and 4 mg/kg bw
Katsnelson [43]	mean 49 nm (laser ablation - naked)	Rats	Liver, bone marrow, spleen, kidney, peripheral blood & skeletal muscle	IP	Repeated dose	10 mg/kg 3 times a week for up to 20 injections	?	RAPD-test	y	Significantly increased evidence of DNA fragmentation in liver, bone marrow, spleen, kidney and peripheral blood cells

The included investigations showed substantial variation in study design including choice of the size of silver particles, animal species, target organs, silver dose, route of administration and the method used to detect genotoxicity.

The AgNP ranged in size from <10 nm (6 studies) up to 200 nm (2 studies - microparticles), with 3 studies testing different AgNP sizes. The majority of studies did not explicitly state whether the AgNP used were naked or capped. When stated, capping/stabilising agents include poly styrene-co-maleic anhydride and PVP. Seven studies used rats and 10 mice. Target organs included bone marrow (7), peripheral blood (4), spleen (2), testes (2), lung (1), skeletal muscle (1) and kidney (1). In addition, one study looked at the impact on the offspring of pregnant test animals.

Of the 17 studies, 6 administered the doses by intraperitoneal injection, 4 by intravenous injection, 1 by inhalation and 6 orally. Most studies (9) gave just a single dose but the remaining 8 studies gave repeated doses of AgNP. For the repeated dosing experiments the duration and frequency varied substantially (Table 3). The doses administered to the animals varied by as much as 5 logs, ranging from 0.01 to 1000 mg/kg. The comet assay was used in 11 investigations, the micronucleus assay in 6, 2 used microscopy of cells in metaphase to look for chromosomal abnormalities and 1 used the randomly amplified polymorphic DNA (RAPD) test.

**Table 3:** Summary multiple dosing regimen

Frequency of dosing	Duration of dosing	Number of studies
Daily	5 days	2
Daily	28 days	2
Alternate days	28 days	1
3 times a week	20 injections	1
Weekly	5 weeks	1

Of the 17 studies, 11 reported evidence of genotoxicity (Tables 1 and 2). Table 1 outlines the studies using in vivo micronucleus or comet assays, which are the basis of the current OECD guidelines for in vivo genotoxicity testing. Table 2 list the studies using other methods. Where multiple cell/tissues were examined and some were found not to show evidence of genotoxicity, we have only referred to the significant findings in the Table. Of the 6 studies that reported not finding evidence of an in vivo genotoxic effect of AgNP, 2 were the first such studies to be reported [10, 16]. Three of the negative studies used lower concentrations (in a single dose) than the most of the other studies (0.01, 0.025 and 0.05 mg/kg - [17]; 0.25 and 1 mg/kg - [18] and 1.5 mg/kg - [16]). While Kim et al. suggested that their results were not statistically significant they did observe a non-significant trend in micronucleus formation in males with increasing dose and an increased effect in females at 2 of the 3 doses [10]. Consequently, mammalian in vivo evidence of genotoxicity was reported in the majority of studies, providing that a sufficient dose of AgNP were administered.

DNA deletions and breakages were reported particularly frequently across the studies, with the most worrying being the detection of large DNA deletions in developing embryos, albeit after a very large oral dose of 500 mg/kg daily for five days in pregnant mice [19]. In addition, a significant risk of DNA damage in spermatozoa was reported by Gromadzka-Ostrowska et al. further raising concerns about damage to future generations [20].

The selected papers were subjected to a reliability assessment and, as can be seen from Table 1, only 2 studies were classed as reliable (with or without restrictions). The principal reason for the ‘not reliable’ classification was the absence of a positive control, which is recommended for both the in vivo mammalian alkaline comet assay and the in vivo mammalian micronucleus test [21, 22]. If the fact that (in the majority of cases) assays generally gave a positive result means that a positive control is not a requirement, then a further 7 studies can be classified as reliable. The two remaining studies (Table 2) could not be rated for quality as they had not used a method for which there are internationally accepted guidelines.

One further paper is worthy of note because it is the only study, so far, to have investigated the possible relationship between DNA damage in humans and silver exposure [23]. These authors investigated evidence of DNA damage in silver jewellery workers who are at risk of inhalation of silver particles. The comet assay was used to investigate DNA damage in mononuclear leukocytes and showed a significant increase in DNA damage in the jewellery workers compared to local controls. While suggestive the findings of the study need to be interpreted with care as there are a number of limitations (including a small number of participants and a lack of direct measurements or estimates of exposure).

We did not find any papers which suggested that in vivo exposure to ionic silver posed a threat of genotoxicity, but those few studies that reported on ionic silver found no effect even from in vitro studies [6].

## Discussion

We have presented the first systematic review of in vivo genotoxicity studies of AgNP in mammalian models. The majority of the identified studies reported evidence of genotoxicity and the negative studies were generally those using single small doses. The potential genotoxic effect was supported by a single human observational study, which reported evidence of the accumulation of severe DNA damage in jewellery workers who were occupationally exposed to silver particles and AgNP. While, as noted by the European Food Safety Authority (EFSA) [24] the studies do not allow a definitive assessment of human genotoxic hazard associated with oral exposure to AgNP, we feel that the results are suggestive that such a risk may exist.

In our review, we found substantial variation in study design including choice of the size of silver particles, animal species, target organs, silver dose, route of administration and the method used to detect genotoxicity. There are also numerous different methodologies for the synthesis of AgNP they can, for example, be produced in a range of sizes and shapes and stabilised with a variety of capping agents; these factors alone make generalisations difficult. In addition, some capping agents and manufacturing contaminants may be toxic in their own right [25], although this does not appear to have been investigated in relation to genotoxic effects

The studies used a range of assay methods, not all of which may comply with the current OECD recommendations. While there has been some debate in the literature on the most appropriate methods for genotoxicity testing of nanoparticles (e.g. Warheit and Donner [26]), recent expert opinions have not suggested a move away from existing protocols [21, 22].

A further complication in relation to applying the findings to oral ingestion is the high proportion of studies administering AgNP via injection (intravenous and intraperitoneal); given that Ag absorption is likely to be lower following oral ingestion it would not be surprising if studies using injected silver over-estimate risk from ingestion. It should be noted, however, that 5 of the 6 studies using oral administration showed statistically significant genotoxic effects, and the one study reporting negative results showed a non-significant increase in DNA damage with increasing dose [10].

The comet assay is classed as an ‘indicator test’ because the measured end point may not always lead to a mutation [27]; this raises the issue of whether or not some of the DNA damage described in the reported studies is likely to be reversible. In one study, with relatively low exposure, the DNA damage did appear to be reversible with time [17]. Clearly, DNA repair mechanisms will reduce the genotoxic potential of AgNP. However, it is not certain how effective these repair mechanisms will be in those tissues such as the brain and testes where bioaccumulation of AgNP has been reported to occur. In addition, it is unlikely that “the large DNA deletions” in developing embryos and “irreversible chromosomal damage” in bone marrow described by Kovvuru et al. [28] are transient (although the dose used in this study was particularly high - 500 mg/kg for 5 days) and the finding of the “accumulation of severe DNA damage” in people occupationally exposed to silver particles certainly suggests that concerns about human safety are real [23]. While it is not possible from this observational study to quantify exposure in the jewellery workers, these findings would suggest that DNA repair mechanisms can be overwhelmed with frequent exposure to silver particles. It is certainly plausible that consumers using colloidal Ag as the primary drinking water disinfectant or workers adding AgNP to ceramic filters in low income countries would have similar or even greater exposure than the jewellery workers. The limited available evidence indicates that health and safety precautions are not strictly adhered to in the production of ceramic filters in low income countries [29].

The wide heterogeneity in study designs makes it difficult to draw substantial conclusions and, especially, to determine a safe daily exposure to AgNP. Without being able to state a safe exposure limit, it is not possible to say if exposure to the colloid Ag or AgNP employed in some current household water treatment practices poses a significant risk to human health. However, given that the use of AgNP and colloidal Ag in household water treatment does not appear to be effective at making drinking water safe [30], there is little value in their use and any risks of genotoxicity are consequently intolerable. Given the likely bioaccumulation especially in brain and testes, people using colloidal Ag as a primary drinking water disinfectant and their offspring may be at high risk of genetic damage. The risks associated with use of AgNP in other matrices, such as ceramic filters, are less clear. There is substantial evidence that AgNP are released from many different supporting matrices [31], but it is not yet possible to say with certainty whether or not such release would be in sufficient concentration to pose a health risk.

## Conclusions

We consider that colloidal Ag should not be promoted as a primary water treatment product as it has little public health benefit and the balance of evidence would certainly suggest that there is at least the possibility of genotoxic and embryotoxic effects. With the current evidence it is not yet possible to determine a safe limit for the oral intake of AgNP or colloidal Ag. Before colloidal Ag or AgNP are used in filter matrices for drinking water treatment, consideration needs to be given to how much silver is likely to be released from the matrix during the life of the filter (eg work by Garboś and colleagues) [32, 33]. There is an urgent need for more research on this topic and particularly on whether people using colloidal Ag as a primary drinking water treatment, working with AgNP in filter manufacture in low income countries or using such impregnated filters have evidence of DNA damage.

## Abbreviations

Ag: Silver
AgNO3: Silver nitrate
AgNP: Silver nanoparticles
CMC: Carboxymethylcellulose
CTAB: Cetylmethylammonium bromide
EFSA: European Food Safety Authority
OECD: Organisation for Economic Co-operation and Development
POU: Point-of-use
PVP: Polyvinylpyrrolidone
RAPD: Randomly amplified polymorphic DNA
TDI: Tolerable daily intake
WHO: World Health Organization
